# The complete chloroplast genome of *Verbascum thapsus* L. (Scrophulariaceae) and its phylogenetic affinities

**DOI:** 10.1080/23802359.2022.2113749

**Published:** 2022-09-02

**Authors:** Yue Zhang, Yun-Hui Guan, Cheng-Zhao Pu, Yi-Jia Xi, Ai-en Tao, Cong-Long Xia

**Affiliations:** a College of Pharmacy, Dali University, Dali, China; b College of Medicine, Lijiang Culture and Tourism College, Lijiang, China

**Keywords:** *Verbascum thapsus*, chloroplast, Illumina sequencing, phylogenetic affinities

## Abstract

*Verbascum thapsus* L. has extensive pharmacological effects, including antioxidative and antineoplastic action, memory improvement and neuroprotection. However, its phylogenetic position is not established in Scrophulariaceae. In this study, we reported the complete chloroplast genome sequence of *V. thapsus* L. for the first time and investigate its phylogenetic relationship in Scrophulariaceae. The assembled chloroplast genome is a circular 153,338 bp sequence, including a large single copy (LSC) region of 84,627 bp, a small single copy (SSC) region of 17,829 bp and a pair of inverted repeats (IRs) of 25,441 bp. The genome contains 135 genes, including 86 protein coding genes, 37 tRNA genes, and eight rRNA genes. The phylogenetic tree showed that *V. thapsus* is closely associated with *V. chinense* and *V. phoeniceum.*

The genus *Verbascum* (Scrophulariaceae) contains about 300 species with medicinal properties, most of which are distributed across Europe and Asia. Currently, there are six *Verbascum* species recognized in China (Süntar et al. 2011; Ntantiso and Jaca 2015; Frezza et al. 2019). Among these species, *Verbascum thapsus* Linnaeus 1753 is widely distributed in southwest China and has been used in ethnic medicine for treatment of osteoporosis and depression (Minru and Yi 2016; Frezza et al. 2019). Until now, however, most studies on *V. thapsus* have concentrated on morphology and phytochemistry rather than molecular evolutionary history (Babamoradi et al. 2018). Chloroplast (cp) genomes are precious resources for phylogenetic studies and plant molecular identification (Wei et al. 2020). With such data, the phylogenetic positions of two *Verbascum* species have been clarified (Bi et al. 2020; He et al. 2020). In this study, we report the complete chloroplast genome sequence of *V. thapsus* and elucidated its phylogenetic position in Scrophulariaceae. This provides the basis for further study on the genetic diversity, phylogenetic relationships, and evolution of *V. thapsus*.

Fresh and young leaves of *V. thapsus* were collected from Lijiang, Yunnan, China (N26°55′33.88″, E100°14′09.02″). A voucher specimen was deposited in the Culture and Tourism College of Yunnan University (www.lywhxy.com, Contact person: Ai-en Tao, and Email: 2515073996@qq.com) under the voucher number: TC01. Total genomic DNA was extracted from fresh leaves using a modified CTAB method (Doyle and Doyle 1987; Yang et al. 2014) and genome sequencing of 150 bp paired-end reads was performed using the Illumina Hiseq 2500 platform by Genesky Biotechnologies Inc. (Shanghai, China). About 4 G data were obtained and assembled with GetOrganelle v1.6.2 (Jin et al. 2020), and annotated with the OGAP pipeline (https://github.com/zhangrengang/ OGAP). The draft annotations were then adjusted manually. The annotated genomic sequence has been submitted to GenBank (accession number: MT012419).

The complete chloroplast genome of *V. thapsus* is 153,338 bp in length with a typical quadripartite structure, which contained a pair of inverted repeat regions (IRa and IRb) of 25,441 bp, and a large single copy (LSC) region of 84,627 bp, a small single copy (SSC) region of 17,829 bp. The content of guanine(G) and cytosine(C) in the whole chloroplast genome is 36.0%. In addition, The cp genome contained 135 genes, including 86 protein coding genes, 37 tRNA genes, and eight rRNA genes.

To investigate its phylogenetic position, we used complete chloroplast genome sequences from *V. thapsus* and 27 other Scrophulariaceae species downloaded from GenBank. The resulting phylogenetic tree was rooted with two *Salvia* species. These chloroplast genome sequences were aligned using MAFFT v.7.471 (Katoh and Standley 2013). Phylogenetic analysis was conducted with maximum likelihood and 1000 bootstrap replicates using IQTree (Nguyen et al. 2015). Bootstrap values were calculated using the in-built UFBoot within IQTree, and the best nucleotide substitution model, GTR + R6, was also estimated in IQTree. The phylogenetic reconstruction revealed *V. thapsus* formed a well-supported clade with two other *Verbascum* species (Figure 1). This relationship will require further phylogenetic studies with additional taxa from *Verbascum* and related genera.

**Figure 1.**
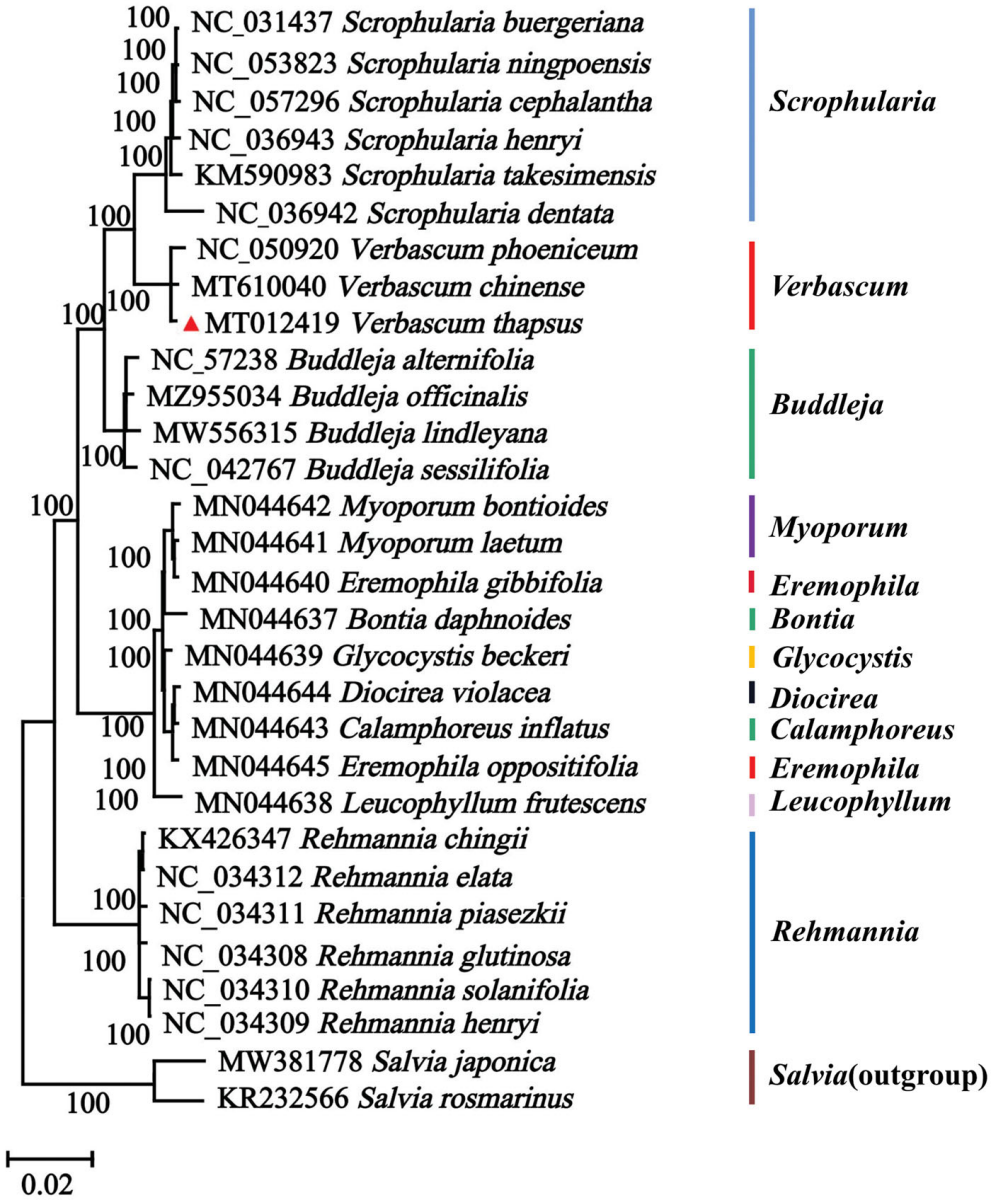
Maximum-likelihood (ML) phylogenetic tree for *V. thapsus* based on 30 complete chloroplast genomes. The numbers on the nodes indicate bootstrap values from 1000 replicates.

In this study, we sequenced and annotated the cp genome of *V. thapsus* and used these data in a phylogenetic analysis. This provides a valuable genomic resource for future research on the evolutionary history and conservation of *Verbascum* species.

## Data Availability

The genome sequence data that support the findings of this study are openly available in GenBank of NCBI at https://www.ncbi.nlm.nih.gov/, under the accession no. MT012419. The associated BioProject, SRA, and Bio-Sample numbers are PRJNA798180, SRR17653644, SAMN25049704, respectively.
